# Editorial: Crosstalk between lung and brain, heart, kidney and vascular system in critical illness

**DOI:** 10.3389/fphys.2024.1516682

**Published:** 2024-11-05

**Authors:** Denise Battaglini, Patricia R. M. Rocco

**Affiliations:** ^1^ Department of Surgical Sciences and Integrated Diagnostics (DISC), University of Genova, Genova, Italy; ^2^ Anesthesia and Intensive Care, IRCCS Ospedale Policlinico San Martino, Genova, Italy; ^3^ Laboratory of Pulmonary Investigation, Carlos Chagas Filho Institute of Biophysics, Federal University of Rio de Janeiro, Rio de Janeiro, Brazil

**Keywords:** crosstalk, lungs, kidney, heart, respiratory failure, ARDS

Crosstalk between the lungs and other organs, such as brain, heart, kidneys, as well as vascular system represents a complex physiologic interaction mediated through cellular signals, soluble mediators, and neurohormonal pathways (Battaglini et al., 2023). This fascinating concept of crosstalk indicates bidirectional communication, encompassing the flow of information and feedback between organs and systems. While organ crosstalk plays an important role in coordinating and maintaining homeostasis through physiological reflexes, it can also contribute to acute or chronic dysfunction in one or more organs (Mrozek et al., 2020). Effective management in such cases often requires a multifaceted approach, including respiratory support, cardiovascular monitoring, renal protection, and anti-inflammatory strategies (Battaglini et al., 2023).

Our Research Topic “Crosstalk between the lungs and the brain, heart, kidneys and vascular system in critical illness” comprises five published articles, which focus on the interplay between lungs, heart, and kidneys. Rocha et al. highlighted the existance of bidirectional communication between the lungs and the heart in acute respiratory distress syndrome (ARDS). ARDS is triggered by an initial injury that initiates a cascade of pathological processes, including damage to lung epithelial and endothelial cells, disruption of the extracellular matrix, immune cell activation, and the release of inflammatory mediators. These changes lead to increased permeability of the alveolar-capillary barrier, resulting in interstitial and alveolar edema, lung collapse, and subsequent hypoxia and hypercapnia. The interplay of sympathoadrenal overstimulation and pro-inflammatory cytokine release results in a vicious cycle, characterized by increased myocardial oxygen demand and release of catecholamines, which is further complicated by diminished contractile function due to inflammation and direct cardiac damage. The use of positive pressure mechanical ventilation (MV) causes pressure overload on the right ventricle (RV) The RV dilates, resulting in raised systolic and diastolic RV pressures. The dilated RV pulls the interventricular septum toward the left ventricle (LV), increasing LV pressure, inducing tricuspid regurgitation, and elevating right atrial (RA) and central venous (CV) pressures. The resulting systemic congestion contributes to organ dysfunction, as the compression of the LV by the dilated RV reduces cardiac output. Together with with shock and hypoxemia, this exacerbates systemic congestion and increases the risk of multi-organ failure (Rocha et al.). This literature review examines the interplay between the heart and lungs in the context of ARDS, highlighting the pathophysiological mechanisms involved, the effects of mechanical ventilation, and the impact of hypoxemia and hypercapnia on cardiac function.

Lung-heart interactions can manifest clinically through electrocardiogram (ECG) changes, as demonstrated by Young et al. They conducted a single-center retrospective cohort study involving 280 patients who underwent lung transplantation for end-stage lung disease. ECG data were collected at different time points: before transplantation, and at 1 week, 1 month, 3 months, and 6 months post-transplant. Significant changes were observed, including reductions in the PR interval, QRS duration, QT interval, QTc interval, and heart rate from pre-transplant to 1-month post-transplant. Notably, the QTc interval showed a significant reduction after transplantation, with no significant changes noted between 1 and 6 months post-transplantat. Patients whose QTc interval decreased by more than 35 ms at 1-month post-transplant had a significantly higher risk of 1-year mortality. These ECG alterations in patients with end-stage lung disease and after lung transplantation may carry important clinical and prognostic implications. Young et al.

Renard et al. conducted a single-center retrospective study on COVID-19 ICU patients who underwent a chest computed tomography (CT) scan within 24 h before or after admission to identify factors associated with lung injury volume. Multivariate analysis revealed that plasma levels of GRP78, an indicator of endoplasmic reticulum stress, were strongly correlated with lung injury volume. Higher GRP78 levels were associated with greater lung injury, whereas lower levels were observed in ICU survivors. Furthermore, interleukin-6 (IL-6) levels were associated with the Sequential Organ Failure Assessment (SOFA) score at admission, demonstrating a link with systemic inflammation, but not directly to lung damage volume. He lack of a significant association between GRP78 and IL-6 levels suggests that endoplasmic reticulum stress and systemic inflammation may operate independently in this context. The study concluded that systemic inflammation is more closely tied to organ failure than lung injury, offering new insights into the pathophysiology of inter-organ interactions Renard et al.

Mechanical ventilation is an independent risk factor for developing acute kidney injury (AKI). In their mini-review, Kumar et al. highlighted the key mechanisms underlying lung-and-kidney interaction. Mechanical ventilation, increases intrathoracic pressure, decreases cardiac output, and causes atrial stretch, which can negatively affect blood flow to the kidneys, leading to renal function impairment. Additionally, positive pressure ventilation triggers neurohormonal pathways that can further compromise kidney function by inducing pre-renal vasoconstriction. This includes the release of antidiuretic hormone, decreased atrial natriuretic peptide, and the activation of renin-angiotensin and sympathetic systems. A meta-analysis by van den Akker et al. confirmed that invasive mechanical ventilation is an independent risk factor, tripling the odds of AKI (van den Akker et al., 2013). In fact, the risk of renal damage in mechanically ventilated patients is alarmingly high, reaching up to 39%. (Vemuri et al., 2022). Ventilator-induced lung injury (VILI) is another critical factor, as the inflammatory mediators released during VILI may cause microvascular dysfunction and endothelial activation, ultimately affecting kidney function. Kumar et al.

Mechanical power (MP) is a key component of mechanical ventilation, reflecting the net injurious effects of ventilation on the lungs. Zhang et al. investigated the relationship between MP, cardiac output and pulmonary blood flow in a pig model of ARDS. Their hypothesis was that MP correlates with both cardiac output and pulmonary blood flow. Pigs were induced with VILI and ARDS and were randomized to receive one of three strategies: open lung approach, ARDS Network high-PEEP/FiO_2_ strategy, and low-PEEP/FiO_2_ strategy. Total MP was calculated based on the energy required to overcome airway resistance and the energy stored in elastic lung tissue. Pulmonary perfusion distribution was assessed with positron emission tomography, comparing regional pulmonary blood flow and MP across three lung regions. The Low-PEEP strategy exhibited a higher overall MP compared to the open-lung group, but cardiac output remained consistent across all groups. Total MP showed a positive correlation with cardiac output and regional pulmonary blood flow across all regions Zhang et al. This study highlights the importance of lung-protective ventilation strategies in critically ill patients to optimize hemodynamic status.

In conclusion, our Research Topic sheds light on the complex mechanisms involved in the crosstalk between the heart, lungs, and kidneys in critical illness. Future research should focus on further elucidating these interactions and developing targeted therapeutic strategies that address the dysfunction of multiple organ systems simultaneously.
